# Exploring in-vitro antioxidant, cytotoxicity, hemolytic, thrombolytic and anticancer potentials of *Ochthochloa compressa* (Forssk.) Hilu

**DOI:** 10.1371/journal.pone.0332194

**Published:** 2025-09-24

**Authors:** Muhammad Atif, Mirza Imran Shahzad, Muhammad Younus Khan, Tahir Maqbool, Jawaria Aslam, Faheem Hadi, Gildardo Rivera, Abdul Rauf

**Affiliations:** 1 Department of Biochemistry & Molecular Biology, IBBB, The Islamia University of Bahawalpur, Pakistan; 2 Faculty of Medicine and Allied Health Sciences, The Islamia University of Bahawalpur, Pakistan; 3 Department of Pharmacy, University of Lahore, Sargodha Campus, Pakistan; 4 Institute of Molecular Biology and Biotechnology, The University of Lahore, Lahore, Pakistan; 5 Department of Physiology and Biochemistry, Cholistan University of Veterinary and Animal Sciences, Bahawalpur, Pakistan; 6 Laboratorio de Biotecnología Farmacéutica, Centro de Biotecnología Genómica, Instituto Politécnico Nacional, México; 7 Department of Pharmaceutical Chemistry, Faculty of Pharmaceutical Sciences Prince of Songkla University, Hat-Yai, Songkhla, Thailand; 8 Drug Delivery System Excellence Center (DDSEC) Pharmaceutical Sciences, Prince of Songkla UniversityHat-Yai, Songkhla, Thailand; University of Buea, CAMEROON

## Abstract

**Background:**

Globally, medicinal plants are found therapeutically very effective against many illnesses. Still there is huge demand of plant based alternate medicines in market. The therapeutic potential of many plants is still unexplored. One such medicinal plant is *Ochthochloa compressa*, a member of family *Poaceae* commonly located in Cholistan desert of Bahawalpur, Pakistan.

**Purpose:**

The purpose of this study is to evaluate some pharmacological properties of plant which include; exploration of cytotoxic, hemolytic, antioxidants, anticancer and thrombolytic phytochemicals from various extracts of *O. compressa*.

**Study Design:**

This study includes *in-vitro* experimental study design.

**Methodology:**

*O. compressa* extracts were prepared on the basis of polarity. The extracts were subjected for screening of phytochemicals and determining the total bioactive contents. Each extract was tested for cytotoxicity, hemolytic, antioxidant, thrombolytic and anticancer potential through cytotoxic and cell viability assays; MTT, morphology, crystal violet, trypan blue and scratch. GCMS analysis of each extract was done for identification of phytocompounds. *In-silico* molecular docking was performed against phytocompounds of EtOAc extract and further studied to visualize interactions between the compounds and coagulation factor XI. Compounds which showed maximum binding affinity were used in ADMET studies.

**Results:**

The phytochemical screening and GCMS analysis revealed various phytochemicals in plant extracts. HET CAM assay revealed that all extracts were non and/or weak irritant except DCM which was moderately irritant. The least hemolytic activity was observed in all extracts, which proved that the plant is non-toxic, non-hemolytic and safe to use. The extracts showed a good antioxidant potential and order was as; MtOH > Aq > *n-*Hex > EtOAc > DCM. Promising anticancer potential was observed by extracts of this plant against HepG2 cell line and order of activity was as; n-Hex > MtOH = Aq = DCM > EtOAc. Notably high thrombolytic potential was observed by all extracts especially from EtOAc (96.2 ± 0.88) which was almost equal to Streptokinase (99.3 ± 0.41). Phytocompounds of EtOAc identified by GCMS analysis showed significant binding affinity with coagulation factor XI protein, and upon evaluation of ADMET profile, all compounds followed Lipinski’s rule of five, being suitable for orally administration.

**Conclusion:**

*O. compressa* extracts are good source of antioxidant, anticancer and thrombolytic agents. The plant is non-toxic and non-hemolytic as well.

## 1 Introduction

Medicinal plants are important part of traditional and modern medical field. They are serving as primary healthcare agents for rural and urban communities. Globally, about 50,000 plant species have been used medicinally, and around 70% of the world population still relies on traditional medical systems for healthcare [1]. *Ochthochloa compressa* is a grassy member of *Poaceae*, commonly located in Cholistan desert of Bahawalpur, Pakistan, locally known as *“Phalwan”* or *“Chhimbar”* and reported to be a good fodder grass for cattle and horses [2,3]. A little research has been reported about *O. compressa* in subcontinent [4]. Grasses are extensively used in traditional healthcare system as they contain biological active compounds; alkaloids, flavonoids and saponins [5]. Alkaloids make the grasses extremely resistant against foreign microorganisms. Flavonoids exhibited anticancer and anti-inflammatory potential and proved helpful to repair oxidative cell damage in animals [6]. Plants are rich sources of antioxidant agents, and there is massive concern to use natural antioxidants for treatment of various diseases like cancer, cardiovascular, diabetes, neurodegenerative, and skin diseases [7]. These diseases are linked with reactive oxygen species (ROS) and can be treated with strong antioxidants. Thus, polyphenols and other phytoconstituents with strong ROS scavenging action are key approaches to treat these diseases [8].

HET-CAM assay is an alternative test used for evaluation of cytotoxicity and anti-inflammatory activities of tested compounds or plant extracts against sensitivity of Chorio Allantoic Membrane (CAM) in order to observe irritation, hemorrhage, blood clotting and vascular lysis in case of damages [9]. Hemolytic activity is used in research and diagnostic applications to find the pathogenicity of pathogens or poisons that can produce health issues. Plant extracts are considered dangerous for erythrocytes if hemolysis ratio is greater than 30% while hemolysis below this ratio is considered protected and non-toxic for humans [10].

Cancer is a fatal disease and affects humans worldwide. It causes many deaths every year in both males and females. Millions of people suffer financial burden due to cancerous conditions in several countries [11]. Liver cancer is one of the most challenging cancers with limited treatment options and poor prognosis. Many advanced techniques enable medical science in improving diagnosis of fatal diseases including liver cancer [12]. Several plant extracts have been proved beneficial against liver cancer cell line through *in-vitro* studies [13]. A thrombus (blood clot) formed in the circulatory system due to hemostatic failure results in vascular obstruction and can lead to severe outcomes in thrombolytic conditions such as acute myocardial or cerebral infarction, potentially resulting in mortality [14]. Common thrombolytic drugs used to break up clots include tissue plasminogen activator (tPA), streptokinase, urokinase, alteplase, and anistreplase [15]. Aspirin and heparin are safe but moderately effective in preventing recurrence and speeding up lysis. Further research will yield fresh perspectives and advance the creation of optimal thrombolytic activity that lead to the maximal coronary arterial thrombolysis with minimal bleeding [16]. The preset study is aimed to explore *O. compressa* for its therapeutic potentials including; cytotoxicity, hemolytic, antioxidant, anticancer and thrombolytic.

## 2 Materials and methods

### 2.1 Collection and identification of the plant

Aerial parts of the *O. compressa* grass were collected from Cholistan desert of Bahawalpur. The botanical identification and authentication of plant specimen was done by the expert of Department of Botany, The Islamia University of Bahawalpur, and assigned a Voucher No. 240/Botany, dated 09-03-2023.

### 2.2 Extraction of the plant

Plant specimen was washed and shade dried. Later, specimen grounded to form fine powder and stored in airtight containers at RT. The 1000grams of dried powdered material of *O. compressa* was dissolved and macerated in each of the solvents; aqueous or distilled water (Aq), dichloromethane (DCM), ethyl acetate (EtOAc), methanol (MtOH) and *n*-hexane (*n*-Hex) separately in air tight containers under continuous shaking and stirring at room temperature (RT) for 15 days. The macerated mixture was filtered thrice with the help of muslin cloth and further filtered using Whatman grade-1 filter paper. Later on, evaporation of filtrate was done in rotary evaporator under reduced pressure (−760 mmHg) and controlled temperature at 40^o^C. The concentrate was air dried and a thick, semisolid and dark brown mass obtained. Finally, dried material was weighed, labeled and stored at RT in the air tight glass bottles [17].

### 2.3 Phytochemical analysis

The primary and secondary metabolites; alkaloids, amino acids, carbohydrates, glycosides, proteins, phenols, flavonoids, lipids, resins, saponins, steroids, tannins and terpenoids were screened through the qualitative analysis of phytochemicals present in *O. compressa* extracts [18].

### 2.4 Determination of total bioactive contents

#### 2.4.1 Total Flavonoid Contents (TFC).

Estimation of flavonoid contents was carried out using aluminum chloride colorimetric method [19]. The standard quercetin solutions; 30, 40, 50, 60, 70, 80, 90, 100 µg/mL were prepared in 96% ethanol. 1 mg/mL solution of each extract was prepared in methanol and 50µl of each extract (1 mg/ml) was mixed with standard quercetin solutions followed by addition of 10 µL of aluminium chloride (10%) solution and 150 µL of ethanol (96%). Then, 10 µL of sodium acetate (1M) was added to the mixture in the 96 well plates. Ethanol was used as a blank. All the reagents were mixed and mixture was incubated at RT in the dark for 40 min. Absorbance was measured at 415nm with a BioTek Synergy HT microplate reader. Total flavonoid contents were expressed as mg Quercetin Equivalents (QE) per gram of dry extract.

#### 2.4.2 Total Phenol Contents (TPC).

Total phenolic contents were estimated using Folin-Ciocalteu method [19]. Gallic acid was used as a standard in various concentrations (0.05–0.5 mg/mL) for establishing a standard calibration curve. Each extract solution was prepared as 1 mg/mL in methanol, and 25 μL of the diluted extract was taken in a test tube and 0.1mL of Folin-Ciocalteu’s reagent was added in it. Then 2.8mL of Na_2_CO_3_ (10%) solution was pooled in the resulting solution and kept in the dark for 30 min. Absorbance was measured at 765nm with a BioTek Synergy HT microplate reader. Total phenolic contents were expressed as mg Gallic Acid Equivalents (GAE) per gram of dry extract.

#### 2.4.3 Total Tannin Contents (TTC).

The total tannin contents were determined using the Folin-Denis spectrophotometric method [20]. The 1 mg of each extract was strongly mixed with 10mL of distilled water with constant stirring and kept at RT for 30 min. The mixture centrifuged and supernatant was collected. Then 2.5mL of extract supernatant was added to a 50mL volumetric flask. Then 1mL of Folin Denis’s reagent and 2.5mL of Na_2_CO_3_ saturated solution was added in the flask. Finally, volume was made up to 50mL and incubated at RT for 90 min. Absorbance was measured at 250nm using an IRMECO UV-Vis spectrophotometer (model U2020). Total tannin contents were expressed as mg of Tannic Acid Equivalent (TAE) per gram dry extract of plant.

### 2.5 Hen’s Egg Test-ChorioAllantoic Membrane (HET CAM) assay

To perform HET CAM, 1 mg/mL solution of each extract was prepared in methanol and 300 µL of each extract solution was used. Test was performed using normal saline for membrane hydration in hens’ egg. Membrane was observed for coagulation, vasoconstriction and hemorrhage for 5 min as described in method [21].

### 2.6 Hemolytic activity

10mL blood was taken from healthy human volunteers and added in a sterile ethylenediaminetetraacetic acid (EDTA) tubes containing screw-top. Blood was centrifuged for 5 min at 850g and upper layer was discarded. Erythrocytes were washed for several times using 10mL of phosphate-buffered saline (PBS) pH 7.4. The washed cells were re-suspended in cold sterilized BPS (20mL) pH 7.4. Each extract solution was prepared as 1 mg/mL in methanol and 975 µL of each extract was added to 25 µL of RBCs solution and incubated at 37^o^C for 60 min. Absorbance of hemoglobin in the supernatant was noted at 540nm. Triton X-100 (0.1%) was used as positive while PBS as negative control. Rate of hemolysis was calculated via given formula [22].


Hemolysis % =(Abs of sample−Abs of−ve control)/Abs of +ve control × 100.

### 2.7 Antioxidant activity

Free radical scavenging activity of *O. compressa* extracts was determined through DPPH (2,2-diphenyl-1-picrylhydrazyl) assay. Ascorbic acid was used as a standard. 1 mg/mL solution of each extract was prepared in methanol. The 0.1mM solution of DPPH reagent was prepared and 90 µL of this solution was put in a 96-well plate. Then 10 µL of each extract was added in the plate and the mixture was incubated for 30 min. Absorbance was measured at 517nm via BioTek Synergy HT micro plate reader [23].

### 2.8 Anti-cancer activity

#### 2.8.1 Sampling of HepG2 cell line.

HepG2 cell line was taken from the cell and tissue culture lab, Center of Research in Molecular Medicine, Institute of Molecular Biology and Biotechnology, The University of Lahore, Pakistan. The cell line was saved in the cylinder containing liquid nitrogen cylinder. The cell line was thawed from the cryo vials when planned for the culturing [24].

#### 2.8.2 Culturing of HepG2 cell line.

The HepG2 cells were thawed and cultured in the T75 flasks in Dulbecco’s Modified Eagle’s Medium (DMEM, high glucose, Caisson’s Lab, USA) containing penicillin, streptomycin (Caisson’s Lab, USA), as well as 10% fetal bovine serum (FBS, Sigma Aldrich, USA), in a moisten incubator at 37°C providing 5% CO_2_. The medium was changed every 2–3days. DMEM medium without FBS was used for treatment [24].

#### 2.8.3 Treatment of plant extracts against HepG2 cell line.

To perform anticancer activity, plant extracts were dissolved in Dulbecco’s Modified Eagle Medium (DMEM) and extracts concentrations were prepared in same medium and then applied to HepG2 cells for 72hrs.

#### 2.8.4 MTT assay.

Assay was performed using 3-(4, 5-dimethylthiazol-2-yl)-2,5-diphenyltetrazolium bromide, in 96-well plate to assess cytotoxic potential of *O. compressa* extracts in HepG2 cells. Cells were cleaned by phosphate buffer saline (PBS). Cells were incubated in 25µl of MTT solution (5 mg/ml) along with 100µl of serum free DMEM medium for 2h. Formazan crystals of purple tint seen and was solubilized in 10% sodium dodecyl sulfate (SDS). The absorbance was measured at 570nm and ratio of cells viability was calculated [25].

#### 2.8.5 Morphology assay.

Cancer cell lines after successful culturing were incubated with IC_50_ dose of plant extracts for 72hrs. Cell morphology of treated cells was observed under the phase contrast microscopy [26].

#### 2.8.6 Crystal violet assay.

Assay was performed to observe cell viability using crystal violet reagent (Sigma Aldrich, USA). HepG2 cells from all experimental groups were collected within 96-wells plate, washed with PBS and culture medium discarded. Crystal violet dye (0.1%) was mixed with ethanol (2%) and added to HepG2 cells in the plate. The cells were incubated with dye for 15 min at RT to get stained. After incubation, dye was removed and cells were washed gently to save them in wells. 100 µL of 1% sodium dodecyl sulfate (SDS) was poured within each well to solubilize crystal violet stain for 10 min. Absorbance of cell suspensions on micro-titer plate was recorded at 595nm by spectrophotometer [27].

#### 2.8.7 Live dead assay.

Ratio of dead cells was measured by trypan blue for detection of live and dead cells. Treated and untreated cells of trial groups were washed thrice by PBS and incubated in trypan blue (In vitrogen Inc., USA) for 5 min. Later, cells were washed thrice with the PBS and observed in microscope. Cells stained with trypan blue were counted as dead [24].

#### 2.8.8 Scratch assay.

Scratch assay was performed in the 6 well cell culture plate, by applying IC_50_ values of plant extracts. A scratch was made in the culture plate, and images were taken at 0 and 24hrs [28].

### 2.9 Thrombolytic activity

Venous blood (5mL) was drawn from 10–15 human volunteers who were not taking anticoagulant and oral contraceptive remedy for 7days. The blood samples were kept in pre-weighed and sterilized 6 separate centrifuge tubes and incubated for 45 min at 37^o^C. After clot formation, fluid was completely discarded from each centrifuge tube. The weight of clot was calculated by subtracting weight of empty tube from clot holding tube. Streptokinase (SK = 15,00,000 I.U) was diluted with sterilized water (5 ml) and shaken for proper mixing. The streptokinase (30000 IU) was used as positive and distilled water (100 µL) as negative controls. 1 mg of each extract solution was prepared in 1mL of methanol and 100 µL of each extract solution was added along with SK to each Eppendorf tube already containing blood clot. The tubes were incubated at 37^o^C for 90 min and examined for lysis of clot and discharged fluid was wasted. The tubes were weighed again to watch weight variation following the clot lysis. The ratio of clot lysis was calculated as per formula [22].


Percentage of clot lysis=(released clot weight/weight of clot)×100

### 2.10 Gas chromatography mass spectrometry analysis

The phytocompounds were identified from the extracts of *O. compressa* via GCMS analysis using Agilent, 6890 series, and Hewlett Packard, 5973 mass selective detector. An HP-5MS column with a length of 30m, a diameter of 250 µL, and a film thickness of 0.25 µL were used to get the top probably separation. The 1% solution of each extract was prepared by diluting in respective solvent. The 1.0 µL volume was taken from each extract and injected at 250^o^C in a split less mode. The helium gas was used as a carrier at constant flow rate of 1.02mL/min. Then temperature was increased slowly, starting at 50-150^o^C and rising by 3^o^C/min, with a 10 min holding time at each temperature. Finally, temperature was set to 300^o^C at 10^o^C/min. The components were recognized using their retention indices. The mass spectrum was interpreted by the National Institute of Standards and Technology database (NIST) [29].

### 2.11 *In*-*silico* studies

#### 2.11.1 Molecular docking.

Molecular docking is highly helpful tool to study computer-aided drug design. Protein molecules such as coagulation factor-XI (F11, PDB ID: 6TS4, Resolution: 1.17Å) was downloaded in PDB format from the Protein Data Bank (PDB). Protein preparation was done in the Discovery Studio 2021 Client. Water molecules and hetatoms were eliminated from protein molecules. Polar hydrogen molecules were then added to protein molecules and the resultant file was stored as a PDB file. Table 11 was used to select secondary metabolites identified in GCMS analysis of EtOAc extract. The structures of secondary metabolites and standard Milvexian were downloaded in SDF format from the PubChem database, and further saved as PDB format using open babel software. PyRx program, in conjunction with Autodock vina, was utilized in order to accomplish the task of docking the ligands to the active site of the targeted enzymes. Then prepared protein molecule was uploaded in PyRx software and macromolecule option was done. Then ligands were added into PyRx through open-babel option. Later, these ligands were minimized and converted to PDBQT format. Then grid box was adjusted in specific dimensions and started the ligand-protein interaction to analyze the binding results. The exhaustiveness parameter that controls extent of the search was chosen as 8, and 9 modes were generated for each ligand. The best ligand pose selection for the receptor was done based on the docking score. The top choice for ligand pose receptor was made on docking score. Finally, Discovery Studio Visualizer was used to analyze interactions occurred among them [22].

#### 2.11.2 ADME analysis.

Analysis of the chosen bioactive compounds through highest binding affinity was carried out via Swiss ADME (Absorption, Distribution, Metabolism, and Excretion) online software (http://www.swissadme.ch/) [30].

#### 2.11.3 Toxicity evaluation.

Toxicity of phytocompounds was virtually determined with online program proTox-3.0 (https://tox.charite.de/) [31].

### 2.12 Statistical analysis

Graph pad prism 8 software was used for statistical analysis, where one way ANOVA for within groups with bonferroni: compare all pairs of columns, was applied with P value ≤0.05.

## 3 Results

### 3.1 Phytochemical analysis

Preliminary phytochemical analysis of *O. compressa* extracts confirmed the existence of primary and secondary metabolites as shown in Table 1.

**Table 1:** Phytochemical screening for primary and secondary metabolites of **O. compressa*.*

Metabolite(s)	Test(s)	Aq	DCM	EtOAc	MtOH	*n*-Hex
Carbohydrates	Molisch	**+++**	**+++**	**+++**	**+++**	**+++**
Amino acids	Ninhydrin	**--**	**++**	**++**	**--**	**--**
Proteins	Biurette	**--**	**++**	**++**	**++**	**--**
Lipids	Saponification	**++**	**--**	**++**	**++**	**--**
Alkaloids	Hager’s Test Wagner’s Test Mayer’s Test	**++**	**++**	**++**	**++**	**++**
Flavonoids	Reaction with NaOH	**++**	**++**	**+++**	**++**	**++**
Glycosides	Keller Kiliani’s	**+++**	**++**	**++**	**--**	**++**
Phenols	Ferric chloride	**++**	**+++**	**++**	**++**	**+++**
Resins	Acetic Anhydride	**–**	**+**	**+**	**–**	**–**
Saponins	Froth	**–**	**+++**	**–**	**–**	**+**
Steroids/ Terpenes	Salkowaski’s	**++**	**+++**	**+++**	**++**	**++**
Tannins	Lead Acetate	**+**	**+**	**+++**	**+**	**+++**

“+” Present and “−” Absent, Aq: aqueous extract, DCM: dichloromethane extract, EtOAc: ethyl acetate extract, MtOH: methanolic extract, n-Hex: n-Hexane extract

### 3.2 Quantitative analysis of total Phenols, Flavonoids and Tannins

Phytochemical constituents like total phenols, flavonoids and tannins were detected in various extracts of *O. compressa* and quantified further. Statistically, it was apparent that each solvent had an ability to extract diverse range of phytocompounds (p < 0.05). The results are summarized in Table 2.

**Table 2:** Quantitative analysis of the phenols, flavonoids and tannins in the *O. compressa* extracts.

Extract(s)	TPC mg GAE/g DE ± SD	TFC mg QE/g DE ± SD	TTC mg TAE/g DE ± SD
Aq	40.16 ± 1.66	7.85 ± 2.85	13.27 ± 3.18
DCM	111.83 ± 3.33	40.71 ± 2.85	11.9 ± 0.9
EtOAc	64.33 ± 0.83	96.42 ± 5.71	15.09 ± 1.36
MtOH	34.33 ± 0.83	35.71 ± 4.5	11.45 ± 1.36
*n*-Hex	201 ± 2.5	82.85 ± 9.28	18.27 ± 2.72

TPC: total phenolic content, TFC: total flavonoid content, TTC: total tannin content

#### 3.2.1 Assessment of total phenolic content (TPC).

The *n*-Hex extract showed the highest TPC (201 ± 2.5 mg GAE/g DE), medium ones; DCM (111.83 ± 3.33 mg GAE/g DE), EtOAc (64.33 ± 0.83 mg GAE/g DE), aqueous (40.16 ± 1.66 mg GAE/g DE) and lowest MtOH (34.33 ± 0.83 mg GAE/g DE) as shown in Table 2.

#### 3.2.2 Assessment of total flavonoid content (TFC).

EtOAc extract showed the highest TFC (96.42 ± 5.71 mg QE/g DE) medium ones; *n*-Hex (82.85 ± 9.28 mg QE/g DE), DCM (40.71 ± 2.85 mg QE/g DE), MtOH (35.71 ± 4.5 mg QE/g DE) and lowest aqueous (7.85 ± 2.85 mg QE/g DE) as shown in Table 2.

#### 3.2.3 Assessment of total tannin content (TTC).

The *n*-Hex extract showed highest TTC (18.27 ± 2.72 mg TAE/g DE), medium ones; EtOAc (15.09 ± 1.36 mg TAE/g DE), aqueous (13.27 ± 3.18 mg TAE/g DE), MtOH (11.45 ± 1.36 mg TAE/g DE) and lowest DCM (11.9 ± 0.9 mg TAE/g DE) as shown in Table 2.

### 3.3 HET-CAM assay

**Fig 1:**
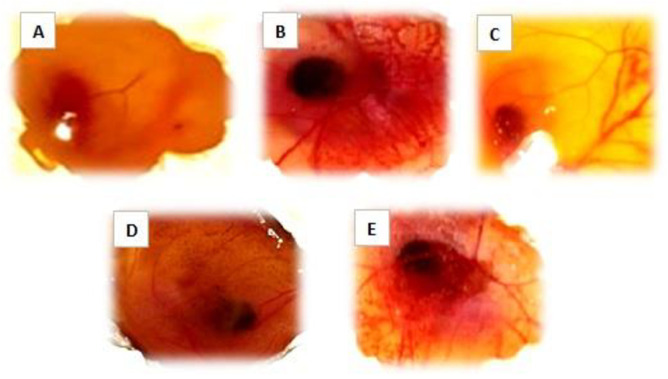
HET CAM activity of *O. compressa* extracts. (A) Aqueous extract = weak irritant. (B) DCM extract = moderate irritant. (C) EtOAc extract = weak irritant. (D) MtOH extract = non-irritant. (E) *n-*Hex extract = weak irritant.

Cytotoxic effect of plant extracts via HET CAM assay revealed that DCM extract was moderate irritant while Aq, EtOAc and *n*-Hex were weak irritant and MtOH extract non irritant. The overall cytotoxic order of extracts was as; MtOH = non irritant, Aq, EtOAc, n-Hex = weak irritant, and DCM = moderate irritant. The effect of extracts producing irritation on membrane in hens’ egg is shown in Fig 1 and Table 3.

**Table 3:** Irritation score and category of *O. compressa* extracts via HET-CAM assay.

Extract(s)	Hemorrhage	Lysis	Coagulation	Cumulative Score	Category
Aq	3	1	0	4	Weak irritant
DCM	1	3	0	4	Moderate irritant
EtOAc	3	1	0	4	Weak irritant
MtOH	0	0	0	0	Non irritant
*n*-Hex	1	0	0	1	Weak irritant

Aq: aqueous extract, DCM: dichloromethane extract, EtOAc: ethyl acetate extract, MtOH: methanolic extract, n-Hex: n-Hexane extract.

### 3.4 Hemolytic activity

**Fig 2:**
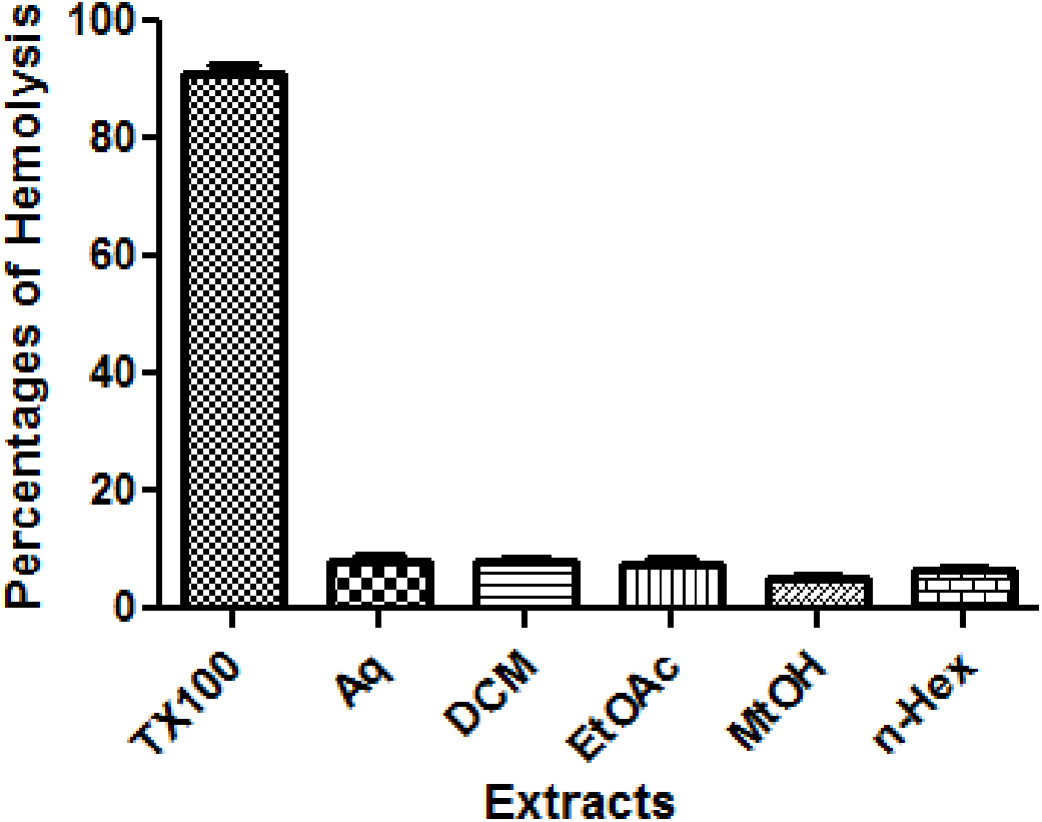
Hemolytic activity of different extracts of *O. compressa.*

Hemolytic activity of *O. compressa* extracts was measured in percentages. Least hemolysis % was observed in all extracts; MtOH (5.053 ± 0.43), *n*-Hex (6.57 ± 0.73), EtOAc (7.68 ± 0.84), DCM (7.84 ± 0.91), and Aq (8.257 ± 0.83) in comparison to Triton X-100 (standard) that showed the highest hemolysis (90.93 ± 1.41). The overall order of hemolytic activity was as; MtOH > *n*-Hex > EtOAc > DCM > Aq. Thus, all the extracts exhibited less than 30% hemolysis of blood that confirmed the extracts are nontoxic, non-hemolytic and safe, as shown in Fig 2 and Table 4.

**Table 4:** Hemolytic activity of *O. compressa* extracts.

Extract(s)	Mean ± SD
Aq	8.25 ± 0.83
DCM	7.84 ± 0.91
EtOAc	7.68 ± 0.84
MtOH	5.05 ± 0.43
*n*-Hex	6.57 ± 0.73
Triton X-100	90.93 ± 1.41

Values were taken as Mean ± SD (n = 3). Triton X-100 was used as standard. Notably results were shown when compared with standard (p < 0.05).

### 3.5 Antioxidant activity

#### 3.5.1 DPPH (1,1-diphenyl-2-picrylhydrazyl) assay.

The results revealed that maximum antioxidant potential was observed in MtOH extract (58.62 ± 0.72 mg AAE/g DE), moderate potential in Aq (21.21 ± 0.06 mg AAE/g DE) and n-Hex (19.1 ± 1.44 mg AAE/g DE), while lower potential was observed in EtOAc (17.66 ± 4.45 mg AAE/g DE) and DCM (16.39 ± 1.62 mg AAE/g DE). The overall order of antioxidant potential was as; MtOH > Aq > *n-*Hex > EtOAc > DCM, as shown in Table 5.

**Table 5:** Antioxidant activity of *O. compressa* extracts.

Extract(s)	mg AAE/g DE ± SD
Aq	21.21 ± 0.06
DCM	16.39 ± 1.62
EtOAc	17.66 ± 4.45
MtOH	58.62 ± 0.72
*n*-Hex	19.1 ± 1.44

Values were taken as Mean ± SD (n=3)

### 3.6 Anti-cancer activity

#### 3.6.1 Cytotoxicity (MTT assay).

**Fig 3:**
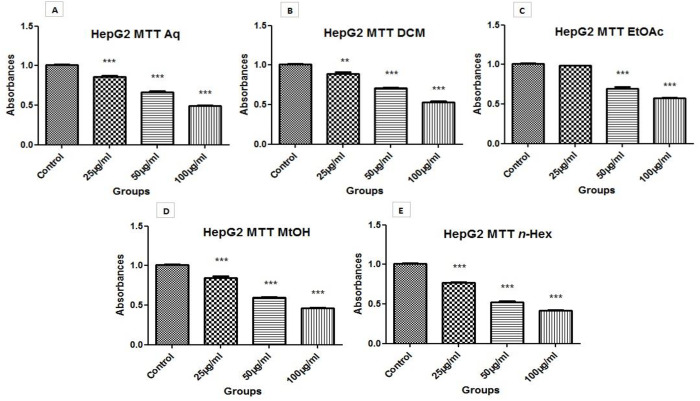
MTT activity evaluation of *O. compressa* extracts. (A) Aqueous. (B) Dichloromethane. (C) Ethyl acetate. (D) Methanol. (E) *n-*Hexane, using three concentrations, 25 µg/ml, 50 µg/ml and 100 µg/ml. Apoptosis of HepG2 cells was assessed between untreated and treated cells. The treated HepG2 cells showed diverse rate of apoptosis as compared to untreated cells. *** is showing significant P value (≤ 0.05).

**Fig 4:**
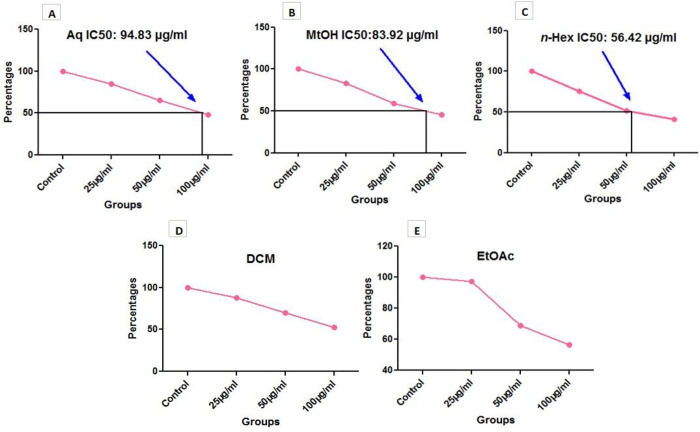
IC_50_ values of *O. compressa* extracts via MTT assay. Three extracts (A) Aqueous. (B) Methanol and (C) *n*-Hexane showed IC_50_ values among three doses, 25 µg/ml, 50 µg/ml and 100 µg/ml, while two extracts (D) Dichloromethane and (E) Ethyl acetate did not showed IC_50_ value. The calculated IC_50_ value of aqueous extract was 94.835 µg/ml, MtOH was 83.924 µg/ml and *n*-Hex was 56.427 µg/ml respectively.

In contrast to control group, a reduced viability was observed in HepG2 cells upon treatment with extracts in different doses; 25 µg/ml, 50 µg/ml and100 µg/ml. The overall cytotoxic effect of extracts was found as; n-Hex > MtOH > Aq > EtOAc > DCM Thus, plant showed cytotoxicity against liver cancer cells. The results of cytotoxicity are presented in Fig 3 and Table 6. Moreover, IC_50_ calculations were performed on the basis of values of MTT assay, as shown in Fig 4.

**Table 6:** Values of cell viability of HepG2 cells treated with plant extracts.

Extract(s)	Control	25 µg/ml	50 µg/ml	100 µg/ml
Aq	1.01 ± 0.00577	0.857 ± 0.0321	0.663 ± 0.0321	0.490 ± 0.490
DCM	1.01 ± 0.00577	0.891 ± 0.0366	0.710 ± 0.0105	0.530 ± 0.0259
EtOAc	1.01 ± 0.00577	0.985 ± 0.00625	0.697 ± 0.0321	0.573 ± 0.0208
MtOH	1.01 ± 0.00577	0.844 ± 0.0399	0.597 ± 0.0162	0.463 ± 0.0115
*n*-Hex	1.01 ± 0.00577	0.767 ± 0.0153	0.524 ± 0.0208	0.417 ± 0.0148

Values were taken as Mean ± SD (n=3).

#### 3.6.2 Morphology assay.

**Fig 5:**
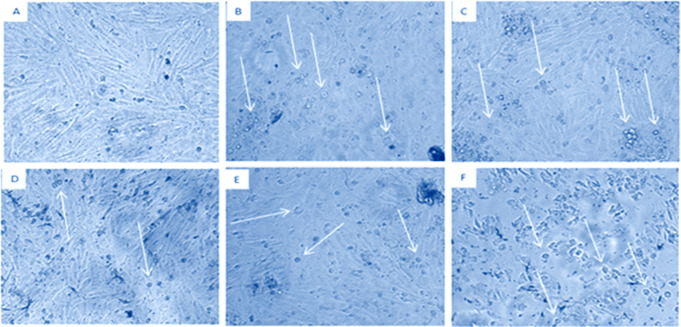
Morphological changes in HepG2 cells exposed to concentration of 100 µg/ml of *O. compressa* extracts for 72hrs. Images were taken using an inverted phase contrast microscope. (A) Untreated HepG2 cells. (B) HepG2 cells treated with Aq extract. (C) HepG2 cells treated with DCM. (D) HepG2 cells treated with EtOAc. (E) HepG2 cells treated with MtOH. (F) HepG2 cells treated with *n*-Hex.

Morphological changes were observed in HepG2 cell line when exposed to concentration of 100 µg/ml of all the extracts for 72hrs. It was observed that cells lost their normal morphology and also lost cell adhesion capacity as compared to control group. Moreover, the majority of cells appeared rounded in the shape as shown in Fig 5. The overall effect of extracts on morphology of the cancer cells was found as; MtOH > Aq > DCM > EtOAc > n-Hex.

#### 3.6.3 Cell viability assay (crystal violet assay).

**Fig 6:**
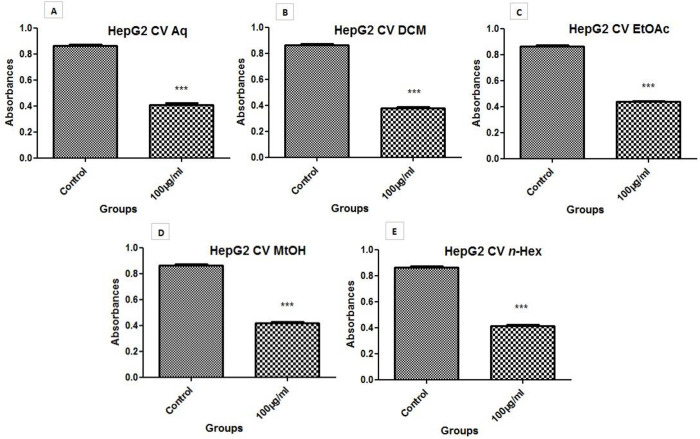
Cell viability evaluation. Cell viability is evaluated using IC_50_ concentration of three extracts (A) Aq. (D) MtOH. (E) *n*-Hex and 100 µg/ml concentration of two extracts (B) DCM and (C) EtOAc) against HepG2 cells for 72hrs. It was observed that treated cells showed less viability as compared to untreated cells. Values were taken as mean ± SD. *** is showing significant P value (≤ 0.05).

HepG2 cell viability was observed in response to plant extracts, by applying their IC_50_ or highest cytotoxic doses; Aq (94.835 µg/ml), MtOH (83.924 µg/ml), *n*-Hex 5(6.427 µg/ml), DCM (100 µg/ml), and EtOAc (100 µg/ml) for 72hrs. A significant reduction of cell viability was observed in treated cells as compared to untreated cells as shown in Fig 6 and Table 7. The overall effect of extracts on cell viability was observed as; DCM > Aq > n-Hex > MtOH > EtOAc.

**Table 7:** Values of cell viability in untreated and treated HepG2 cells.

Extract(s)	Control	100 µg/ml
Aq	0.863 ± 0.0208	0.407 ± 0.0306
DCM	0.863 ± 0.0208	0.380 ± 0.0100
EtOAc	0.863 ± 0.0208	0.437 ± 0.0147
MtOH	0.863 ± 0.0208	0.421 ± 0.0168
*n*-Hex	0.863 ± 0.0208	0.415 ± 0.0150

Values were taken as Mean ± SD (n=3).

#### 3.6.4 Live dead assay (trypan blue assay).

**Fig 7:**
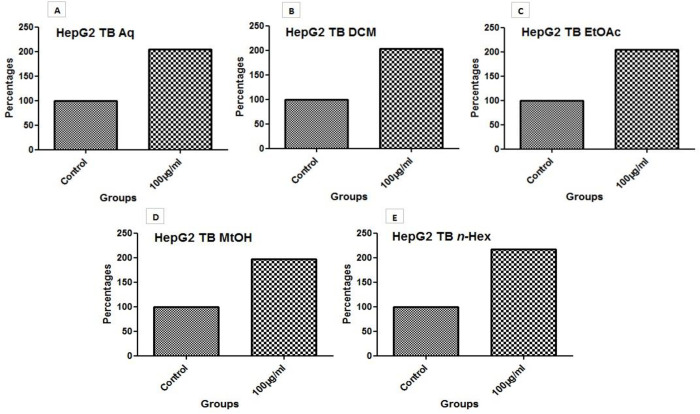
Live dead assay evaluation of extracts Aq, MtOH, *n*-Hex using their IC_50_ concentration while DCM and EtOAc using 100 µg/ml concentration. The death rate of HepG2 cells was increased after treating plant extracts with trypan blue as compared to untreated cells. (A) Untreated and treated cells with Aq extract. (B) Untreated and treated cells with DCM. (C) Untreated and treated cells with EtOAc extract. (D) Untreated and treated cells with MtOH. (E) Untreated and treated cells with *n*-Hex.

IC_50_ doses of three extracts (Aq, MtOH, *n*-Hex), and 100 µg/ml dose of two extracts (DCM and EtOAc) were used to estimate live dead cells in HepG2 cell line. As a result, marked increase was seen in number of dead cells in treated groups as compared to untreated cells as shown in Fig 7. The overall effect of extracts on live dead cells was observed as; n-Hex > Aq = EtOAc > DCM > MtOH.

#### 3.6.5 Scratch assay.

**Fig 8:**
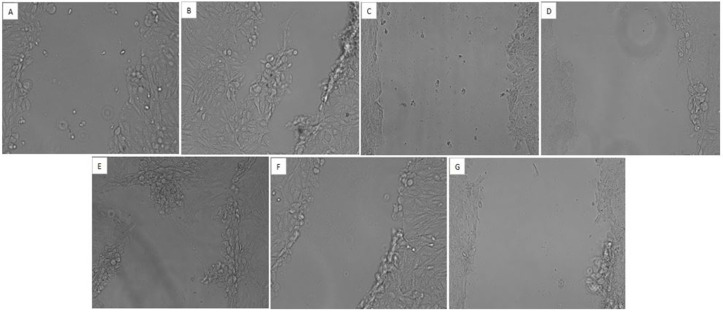
Level of migration of cells through *in-vitro* scratch assay. HepG2 cells grown-up in 6-well plate were treated with *O. compressa* extracts. Images were taken at 0 and 24hrs following treatment via phase-contrast microscope. The migration rate was measured by quantifying the whole distance that cells moved from the edge towards the centre of scratch. (A) Untreated cells at 0hr. (B) untreated cells at 24hrs. (C) MtOH treated cells at 24hrs. (D) DCM treated cells at 24hrs. (E) Aq treated cells at 24hrs. (F) EtOAc treated cells at 24hrs. (G) *n*-Hex treated cells at 24hrs.

HepG2 cell culture plate was observed for scratch movement followed by 24hrs exposure to the extracts. There was found a restricted movement of the cancer cells. While some extracts disrupted the whole cell line and lead to the destruction of cancer cells. Moreover, extracts also prevented cell migration towards the centre of wound point in treated cells whereas scratch space was filled easily by cancer cells in control group as shown in Fig 8. The overall effect of extracts in scratch assay was found as; n-Hex = DCM > MtOH > EtOAc > Aq.

### 3.7 Thrombolytic activity

**Fig 9:**
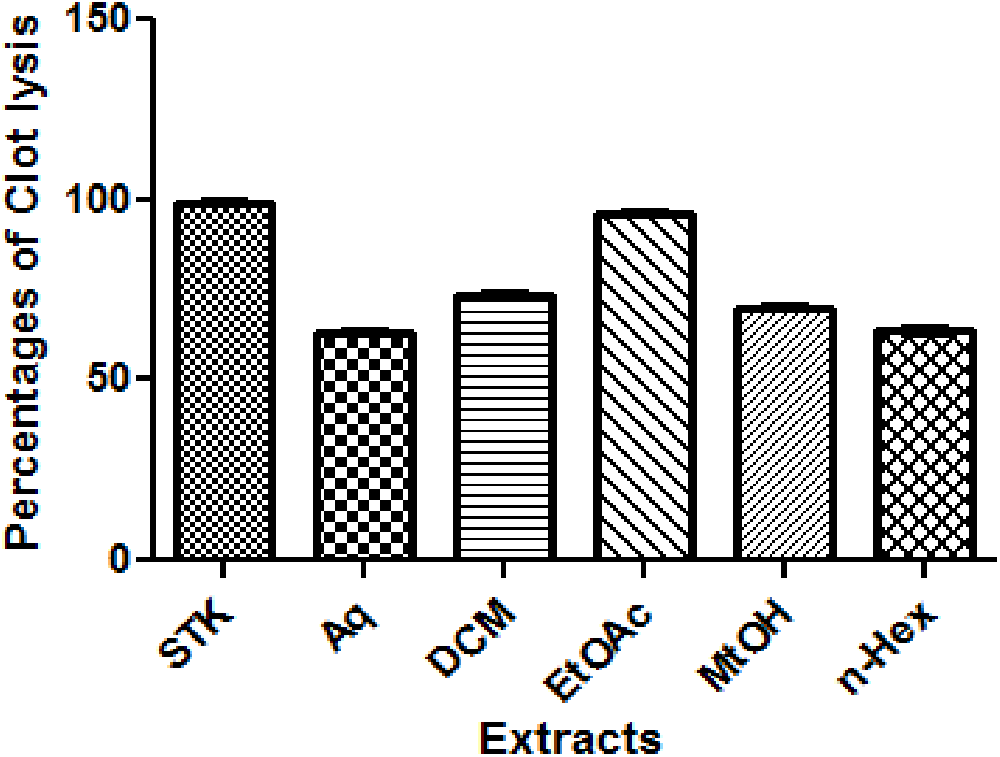
Thrombolytic activity of different extracts of *O. compressa.*

Thrombolytic activity of *O. compressa* extracts was measured in percentages. The uppermost thrombolysis % was observed in EtOAc (96.2 ± 0.88), almost similar to the Streptokinase that exhibited the highest thrombolysis (99.3 ± 0.41). A significant level of thrombolysis % was also observed in remaining extracts; DCM (73.7 ± 0.77), MtOH (69.8 ± 0.82), *n*-Hex (63.7 ± 0.87), and Aq (63.3 ± 0.87). The overall order of thrombolytic potential of the extracts was found as; EtOAc > DCM > MtOH > *n*-Hex > Aq, and presented in Fig 9 and Table 8.

**Table 8:** Thrombolytic activity of *O. compressa* extracts.

Extract(s)	Mean ± SD
Aq	63.3 ± 0.87
DCM	73.7 ± 0.77
EtOAc	96.2 ± 0.88
MtOH	69.8 ± 0.82
*n*-Hex	63.7 ± 0.87
Streptokinase	99.3 ± 0.41

Values were taken as Mean ± SD (n = 3). Streptokinase (SK) was used as standard. Notably results were shown when compared with standard (p < 0.05).

### 3.8 Gas chromatography mass spectroscopy analysis

**Fig 10:**
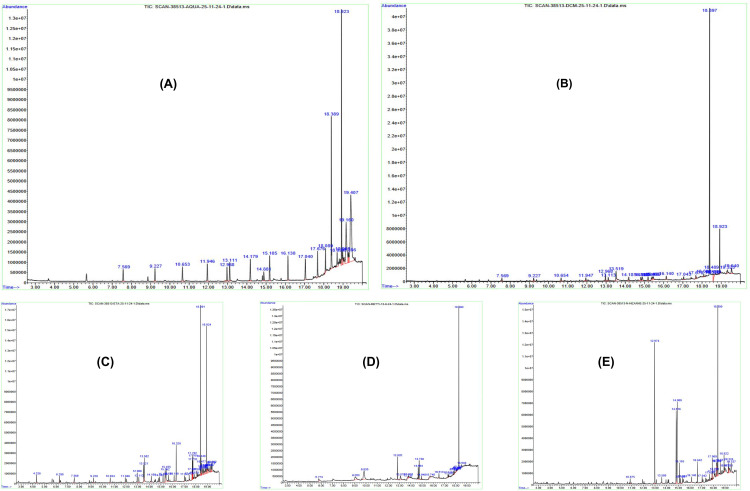
The GCMS chromatograms of five extracts of *O. compressa.* (A) Aqueous. (B) Dichloromethane. (C) Ethyl Acetate. (D) Methanol. (E) n-Hexane.

GCMS analysis of *O. compressa* extracts was performed and various phytocompounds were identified from their chromatograms by comparing; peak area %, retention time and patterns of mass spectral disintegration of the known compounds available in the National Institute of Standards and Technology library (NIST). The GCMS chromatograms of five extracts are expressed in Fig 10.

GCMS chromatograms of Aq extract revealed 13 different phytocompounds as shown in Table 9.

**Table 9:** Compounds identified in the GCMS analysis of Aq extract*.*

No.	RT (min)	PA (%)	Compound Name	MF	MW (g/mol)
1	7.570	1.59	3-Isopropoxy-1,1,1,7,7,7-hexamethyl-3,5,5-tris(trimethylsiloxy)tetrasiloxane	C_18_H_52_O_7_Si_7_	577.2
2	10.654	1.94	2-(2’,4’,4’,6’,6’,8’,8’-Heptamethyltetrasiloxan-2’-yloxy)-2,4,4,6,6,8,8,10,10-nonamethylcyclopentasiloxane	C_16_H_48_O_10_Si_9_	653.3
3	12.966	1.68	Tridecanoic acid, methyl ester	C_14_H_28_O_2_	228.37
4	13.110	2.32	Trisiloxane, 1,1,1,5,5,5-hexamethyl-3,3-bis[(trimethylsilyl)oxy]-	C_12_H_36_O_4_Si_5_	384.84
5	14.883	1.30	Methyl tetradecanoate	C_15_H_30_O_2_	242.4
6	15.184	3.16	3-Butoxy-1,1,1,7,7,7-hexamethyl-3,5,5-tris(trimethylsiloxy)tetrasiloxane	C_19_H_54_O_7_Si_7_	591.2
7	17.039	2.76	N-Benzyl-N-ethyl-p-isopropyl benzamide	C_19_H_23_NO	281.4
8	18.090	2.11	3,6-Dioxa-2,4,5,7-tetrasilaoctane, 2,2,4,4,5,5,7,7-octamethyl-	C_10_H_30_O_2_Si_4_	294.68
9	18.391	11.57	Bis(2-ethylhexyl) phthalate	C_24_H_38_O_4_	390.6
10	18.696	1.92	Cyclohexanone, 2-[2-nitro-1-(2-naphthyl)ethyl]-	C_18_H_19_NO_3_	297.3
11	18.922	18.53	Fumaric acid, 2-ethylhexyl hexyl ester	C_18_H_32_O_4_	312.4
12	19.162	10.81	Indigo	C_16_H_10_N_2_O_2_	262.26
13	19.409	24.10	Phosphonic acid, [1-(1,1-dimethylethyl)-4,4-dimethyl-1,2-pentadienyl]-	C_11_H_21_O_3_P	232.26

RT: retentiontab time, PA: Peak area, MF: molecular formula, MW: molecular weight.

The GCMS chromatograms of DCM extract revealed 15 phytocompounds as shown in Table 10.

**Table 10:** Compounds identified in the GCMS analysis of DCM extract.

No.	RT (min)	PA (%)	Compound Name	MF	MW (g/mol)
1	7.570	0.78	Trisiloxane, 1,1,1,5,5,5-hexamethyl-3,3-bis[(trimethylsilyl)oxy]-	C_12_H_36_O_4_Si_5_	384.84
2	9.227	0.81	2-(2’,4’,4’,6’,6’,8’,8’-Heptamethyltetrasiloxan-2’-yloxy)-2,4,4,6,6,8,8,10,10-nonamethylcyclopentasiloxane	C_16_H_48_O_10_Si_9_	653.3
3	10.654	0.86	3-Isopropoxy-1,1,1,7,7,7-hexamethyl-3,5,5-tris(trimethylsiloxy)tetrasiloxane	C_18_H_52_O_7_Si_7_	577.2
4	12.970	1.57	Tridecanoic acid, methyl ester	C_14_H_28_O_2_	228.37
5	13.518	2.94	n-Hexadecanoic acid	C_16_H_32_O_2_	256.42
6	14.813	0.90	11-Octadecenoic acid, methyl ester	C_19_H_36_O_2_	296.5
7	14.883	1.29	Hexadecanoic acid, methy ester	C_17_H_34_O_2_	270.5
8	15.188	0.97	3-Butoxy-1,1,1,7,7,7-hexamethyl-3,5,5-tris(trimethylsiloxy)tetrasiloxane	C_19_H_54_O_7_Si_7_	591.2
9	15.406	1.01	Trans-13-Octadecenoic acid	C_18_H_34_O_2_	282.5
10	15.46	1.18	9,12-Octadecadienoic acid (Z,Z)	C_18_H_32_O_2_	280.4
11	18.395	48.94	Bis(2-ethylhexyl) phthalate	C_24_H_38_O_4_	390.6
12	18.527	3.52	3-Quinolinecarboxylic acid, 6,8-difluoro-4-hydroxy-, ethyl ester	C_12_H_9_F_2_NO_3_	253.2
13	18.609	2.04	Methyl 9-hexadecenoate	C_17_H_32_O_2_	268.4
14	18.922	6.16	Carbonic acid, propargyl 2-ethylhexyl ester	C_12_H_20_O_3_	212.28
15	19.322	1.39	9-Octadecenamide, (Z)-	C_18_H_35_NO	281.5

RT: retention time, PA: Peak area, MF: molecular formula, MW: molecular weight.

GCMS chromatograms of EtOAc extract showed 14 phytocompounds as shown in Table 11.

**Table 11:** Compounds identified in the GCMS analysis of EtOAc extract.

No.	RT (min)	PA (%)	Compound Name	MF	MW (g/mol)
1	4.326	1.4	1,2,3-Propanetriol, 1-acetate	C_5_H_10_O_4_	134.13
2	12.970	1.65	12-methyltridecanoic acid	C_14_H_28_O_2_	228.37
3	13.523	3.35	n-Hexadecanoic acid	C_16_H_32_O_2_	256.42
4	13.580	4.18	Tetradecanoic acid, ethyl ester	C_16_H_32_O_2_	256.42
5	14.883	1.31	Methyl pentadecanoate	C_16_H_32_O_2_	256.42
6	15.361	2.14	Heptadecanolide	C_17_H_32_O_2_	268.4
7	15.456	5.71	9,12-Octadecadienoic acid (Z,Z)	C_18_H_32_O_2_	280.4
8	15.654	1.34	Neophytadiene	C_20_H_38_	278.5
9	17.43	0.59	4,8,12,16-Tetramethylheptadecan-4-olide	C_21_H_40_O_2_	324.5
10	17.702	3.18	9-Octadecenoic acid (Z)-, 2-hydroxyethyl ester	C_20_H_38_O_3_	326.51
11	17.76	2.39	Pinolenic acid	C_18_H_30_O_2_	278.4
12	17.871	0.43	n-Propyl 9,12,15-octadecatrienoate	C_21_H_36_O_2_	320.5
13	18.391	16.9	1,2-Benzenedicarboxylic acid, dibutyl ester	C_16_H_22_O_4_	278.34
14	19.326	1.38	9-Octadecenamide, (Z)-	C_18_H_35_NO	281.5

RT: retention time, PA: Peak area, MF: molecular formula, MW: molecular weight.

The GCMS chromatograms of MtOH extract expressed 17 compounds as shown in Table 12.

**Table 12:** Compounds identified in the GCMS analysis of MtOH extract.

No.	RT (min)	PA (%)	Compound Name	MF	MW (g/mol)
1	5.773	2.99	4-Vinylphenol	C_8_H_8_O	120.15
2	9.835	14.02	1-Isopropoxy-2,2,3-trimethylaziridine (sin)	C_8_H_17_NO	129.205
3	12.82	8.66	Hexadecanoic acid, methyl ester	C_17_H_34_O_2_	270.5
4	13.079	4.38	4-(1-Hydroxyallyl)-2-methoxyphenol	C_10_H_12_O_3_	180.2
5	13.738	2.46	n-Hexadecanoic acid	C_16_H_32_O_2_	256.42
6	14.664	4.7	cis-13-Octadecenoic acid, methyl ester	C_19_H_36_O_2_	296.5
7	14.749	8.55	9,12-Octadecadienoic acid (Z,Z)-, methyl ester	C_19_H_34_O_2_	294.5
8	14.948	1.24	9,12,15-Octadecatrienoic acid, methyl ester, (Z,Z,Z)-	C_19_H_32_O_2_	292.5
9	15.745	3.37	Oleic Acid	C_18_H_34_O_2_	282.5
10	16.513	1.8	Methyl 18-methylnonadecanoate	C_21_H_42_O_2_	326.6
11	17.318	1.33	4,8,12,16-Tetramethylheptadecan-4-olide	C_21_H_40_O_2_	324.5
12	17.387	0.7	Pentadecanal	C_15_H_30_O	226.4
13	17.823	1.0	Docosanoic acid, methyl ester	C_23_H_46_O_22_	354.6
14	17.887	1.09	1,13-Tetradecadiene	C_14_H_26_	194.36
15	18.147	0.34	Octadecane, 1-iodo-	C_18_H_37_I	380.4
16	18.29	32.77	Bis(2-ethylhexyl) phthalate	C_24_H_38_O_4_	390.6
17	18.538	0.92	Tetracosanoic acid, methyl ester	C_25_H_50_O_2_	382.7

RT: retention time, PA: Peak area, MF: molecular formula, MW: molecular weight.

The GCMS chromatograms of *n*-Hex extract revealed 20 phytocompounds as shown in Table 13.

**Table 13:** Compounds identified in the GCMS analysis of *n*-Hex extract.

No.	RT (min)	PA (%)	Compound Name	MF	MW (g/mol)
1	10.876	0.8	Methyl tetradecanoate	C_15_H_30_O_2_	242.4
2	12.974	21.49	Hexadecanoic acid, methyl ester	C_17_H_34_O_2_	270.5
3	13.58	0.88	Undecanoic acid, ethyl ester	C_13_H_26_O_2_	214.34
4	14.817	11.32	11-Octadecenoic acid, methyl ester	C_19_H_36_O_2_	296.5
5	14.907	20.54	Linoelaidic acid	C_18_H_32_O_2_	280.4
6	15.105	2.99	9,12,15-Octadecatrienoic acid, methyl ester, (Z,Z,Z)-	C_18_H_30_O_2_	278.4
7	15.184	0.90	2-(2’,4’,4’,6’,6’,8’,8’-Heptamethyltetrasiloxan-2’-yloxy)-2,4,4,6,6,8,8,10,10-nonamethylcyclopentasiloxane	C_16_H_48_O_10_Si_9_	653.3
8	15.361	0.73	9-Nonadecene	C_19_H_38_	266.5
9	16.140	1.09	3-Isopropoxy-1,1,1,7,7,7-hexamethyl-3,5,5-tris(trimethylsiloxy)tetrasiloxane	C_18_H_52_O_7_Si_7_	577.2
10	16.643	3.28	Heptadecanoic acid, 16-methyl-, methyl ester	C_18_H_36_O_2_	284.5
11	17.043	0.93	N-Benzyl-N-ethyl-p-isopropyl benzamide	C_19_H_23_NO	281.4
12	17.888	0.6	Methyl myristoleate	C_15_H_28_O_2_	240.38
13	17.908	2.17	Methyl 18-methylnonadecanoate	C_21_H_42_O_2_	326.6
14	18.283	2.03	Pentadecanoic acid, methyl ester	C_16_H_32_O_2_	256.42
15	18.316	3.6	A-Norcholestan-3-one, 5-ethenyl-, (5.beta.)-	C_28_H_46_O	398.7
16	18.391	15.6	Bis(2-ethylhexyl) phthalate	C_24_H_38_O_4_	390.6
17	18.613	1.45	Methyl 18-methylicosanoate	C_22_H_44_O_2_	340.6
18	18.922	2.02	Carbonic acid, propargyl 2-ethylhexyl ester	C_12_H_20_O_3_	212.28
19	19.322	2.06	9-Octadecenamide, (Z)-	C_18_H_35_NO	281.5
20	19.536	2.44	Spiro[androst-5-ene-17,1’-cyclobutan]-2’-one, 3-hydroxy-, (3.beta.,17.beta.)-	C_22_H_32_O_2_	328.5

RT: retention time, PA: Peak area, MF: molecular formula, MW: molecular weight.

Bis(2-ethylhexyl) phthalate was commonly found in extracts; Aq*,* DCM, MtOH and *n*-Hex while 3-Isopropoxy-1,1,1,7,7, 7-hexamethyl-3,5,5-tris(trimethylsiloxy)tetrasiloxane was alike in extracts; Aq*,* DCM and *n*-Hex. The n-Hexadecanoic acid was similar in; DCM*,* EtOAc and MtOH. The 12-methyltridecanoic acid was common in; Aqua*,* DCM*,* EtOAc while 11-Octadecenoic acid, methyl ester was alike in DCM and *n*-Hex. The 2-(2’,4’,4’,6’,6’,8’,8’-Heptamethyltetrasiloxan-2’-yloxy)-2,4,4,6,6,8,8,10,10-nonamethylcyclopentasiloxane was alike in; Aq*,* DCM and *n*-Hex. N-Benzyl-N-ethyl-p-isopropyl benzamide was common in Aq and *n*-Hex. Methyl 18-methylnonadecanoate was similar in MtOH and *n*-Hex while Tridecanoic acid, methyl ester was alike in Aq and DCM extracts. 9,12-Octadecadienoic acid (Z,Z) was similar in; DCM and EtOAc whereas Methyl pentadecanoate was common in EtOAc and *n*-Hex.

### 3.9 *In-silico* studies

#### 3.9.1 Molecular docking studies.

**Fig 11:**
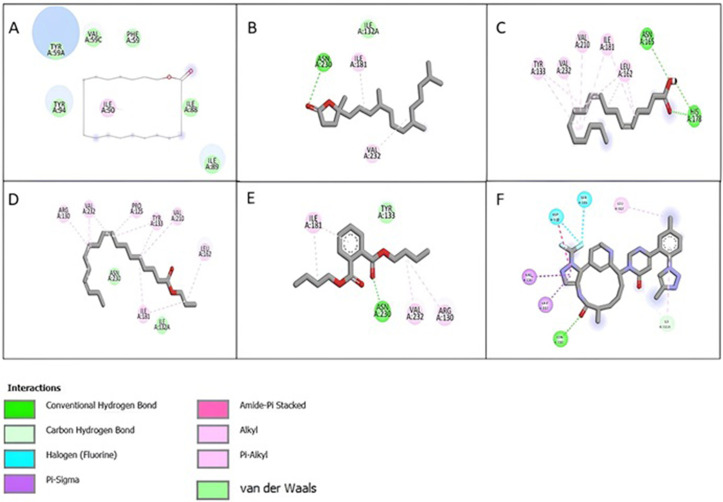
2D structures of interactions of compounds having maximum binding affinity with coagulation factor XI. (A) Heptadecanolide. (B) 4,8,12,16-Tetramethylheptadecan-4-olide. (C) Pinolenic. (D) n-Propyl 9,12,15-octadecatrienoate. (E) 1,2-Benzenedicarboxylic acid, dibutyl ester (F) Milvexian.

The molecular docking was done for the phytocompounds identified by GCMS analysis of EtOAc extract against coagulation factor XI (PDB ID: 6TS4) that revealed a range of binding affinities, with Milvexian, a known FXIa inhibitor used as standard, showing the strongest binding affinity at −9.3 kcal/mol. This result confirms Milvexian’s high potency and validates the docking protocol. Among the tested natural compounds, Heptadecanolide demonstrated the most promising binding affinity (−7.3 kcal/mol), indicating good potential as a lead compound for further thrombolytic research. Although not as potent as Milvexian, its relatively strong interaction with FXIa suggests possible inhibitory activity worth exploring in future *in vitro* or *in vivo* studies. Some compounds such as; Pinolenic acid and 4,8,12,16-Tetramethylheptadecan-4-olide, exhibited moderate binding affinities (−5.6 kcal/mol), implying some degree of interaction but likely insufficient as standalone inhibitors. The majority of the remaining compounds showed weak binding (around −4.4 to −4.9 kcal/mol), suggesting low or non-specific interactions with FXIa. These findings help prioritize compounds for further validation and highlight the Heptadecanolide as a candidate with the greatest potential among the natural constituents of the extract. The 2D structures of interactions of compounds having maximum binding affinity with coagulation factor XI and binding results are expressed in Fig 11 and Table 14.

**Table 14:** Binding affinities of phytocompounds of EtOAc extract with coagulation factor XI.

No.	Compound Name	BA (kcal/mol)
1	1,2,3-Propanetriol, 1-acetate	−4.9
2	12-methyltridecanoic acid	−4.5
3	n-Hexadecanoic acid	−4.6
4	Tetradecanoic acid, ethyl ester	−4.4
5	Methyl pentadecanoate	−4.6
6	Heptadecanolide	−7.3
7	9,12-Octadecadienoic acid (Z,Z)	−4.9
8	Neophytadiene	−4.8
9	4,8,12,16-Tetramethylheptadecan-4-olide	−5.6
10	9-Octadecenoic acid (Z)-, 2-hydroxyethyl ester	−5
11	Pinolenic acid	−5.6
12	n-Propyl 9,12,15-octadecatrienoate	−5.3
13	1,2-Benzenedicarboxylic acid, dibutyl ester	−5.3
14	9-Octadecenamide, (Z)-	−5.2
15	Milvexian	−9.3

BA: Binding affinity.

#### 3.9.2 ADME analysis.

**Fig 12:**
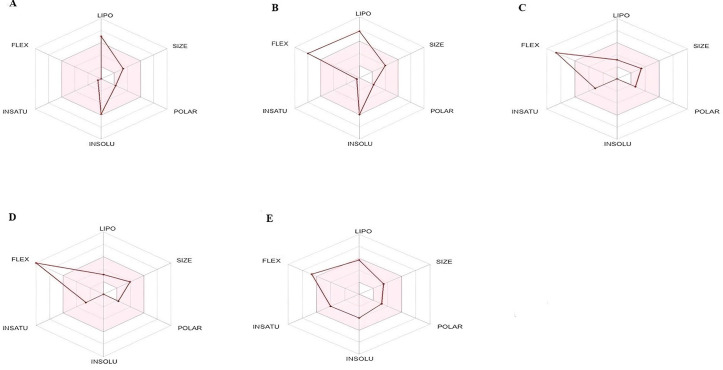
Bioavailability radar of selected phytocompounds. (A) Heptadecanolide. (B) 4,8,12,16-Tetramethylheptadecan-4-olide. (C) Pinolenic. (D) n-Propyl 9,12,15-octadecatrienoate. (E) 1,2-Benzenedicarboxylic acid, dibutyl ester.

Five phytocompounds were chosen from GCMS analysis of EtOAc extract on the basis of highest binding affinity (best docking score). Compounds were further subjected for the ADME analysis via SWISS ADME online software that provided information regarding pharmacokinetics, physicochemical properties and drug similarity of the selected phytocompounds with the best docking scores. Lipinski’s rule of five stated when any phytocompound or drug fails to obey two or more rule; it might consider a non-oral drug or phytoconstituent. In present study, ADME analysis revealed that 3 phytocompounds of EtOAc extract violate one rule and 2 compounds violate zero rules as shown in Table 15. All selected phytocompounds are appropriate and suitable for oral administration as holding orally active drug likeness features. Oral drug delivery system provides wonderful safety, patient compliance, escaping of pain, and has a range of advantages over different routes of drug administration system. Table 15 described the diverse physicochemical properties; molecular weight, pharmacokinetic behavior, lipophilicity, no. of bond rotations, no. of hydrogen bond donor and acceptor of the chosen and analyzed phytocompounds. Fig 12 described bioavailability radar of the selected compounds from EtOAc extract respectively.

**Table 15:** Lipinski’s rule (LR) of five and solubility of the best docked phytocompounds.

No.	Best docked compounds	HBD	HBA	MW	Lipophilicity	M.R	L.R
1	Heptadecanolide	0	2	268.43	4,04	83.00	Yes; 0 violation
2	4,8,12,16-Tetramethylheptadecan-4-olide	0	2	324.54	4.96	102.27	Yes; 1 violation
3	Pinolenic acid	1	2	278.43	5.66	88.99	Yes; 1 violation
4	n-Propyl 9,12,15-octadecatrienoate	0	2	320.51	6.53	102.92	Yes; 1 violation
5	1,2-Benzenedicarboxylic acid, dibutyl ester	0	4	278.34	3.43	77.84	Yes; 0 violation

HBD; hydrogen bond donor, MW; molecular weight, Vn; violation, HBA; hydrogen bond acceptor, MR; molar refractivity. LR; Lipinski’s rule.

#### 3.9.3 Toxicity evaluation.

Five phytocompounds were selected from GCMS analysis of EtOAc extract that exhibited maximum binding affinity (best docking score). Compounds were further analyzed for toxicity analysis using PROTOX online software that provided information regarding; cytotoxicity, carcinogenicity, hepatotoxicity, predicted LD_50,_ predicted toxicity class, mutagenicity, immunotoxicity of the chosen phytocompounds with the best docking scores. All phytocompounds showed the negative results in hepatotoxicity, mutagenicity, immunotoxicity and cytotoxicity. Only 2 compounds; n-Propyl 9,12,15-octadecatrienoate and 1,2-Benzenedicarboxylic acid, dibutyl ester, showed positive results in carcinogenicity. Three phytocompounds showed toxicity class 5. The results of toxicity evaluation of five compounds from EtOAc extract are expressed in Table 16.

**Table 16:** Toxicity evaluation of the selected phytocompounds from EtOAc extract.

Compound Name	Predicted LD_50_ (mg/kg)	Predicted toxicity class	Hepatotoxicity	Carcinogenicity	Mutagenicity	Immunotoxicity	Cytotoxicity
Heptadecanolide	5000	5	_	_	_	_	_
4,8,12,16-Tetramethylheptadecan-4-olide	4400	5	_	_	_	_	_
Pinolenic acid	10000	6	_	_	_	_	_
n-Propyl 9,12,15-octadecatrienoate	20000	6	_	+	_	_	_
1,2-Benzenedicarboxylic acid, dibutyl ester	3474	5	_	+	_	_	_

(+): Toxic, (-): Not Toxic. LD: lethal dose.

## 4 Discussion

*O. compressa* has been explored in various researches and found therapeutically effective against diseases [32]. The grains of *O. compressa* were used as medicine [33]. The plant has been used for skin wound healing as well as treatment of rheumatism [34,35]. In current study, various therapeutic potentials of *O. compressa* have been highlighted. The cytotoxicity of *O. compressa* was tested via HET-CAM assay and the activity is seen in following order; MtOH = non irritant; Aq, EtOAc, *n*-Hex = weak irritant, and DCM = moderate irritant. According to the results, MtOH extract is non-irritant. While the remaining three extracts (Aq, EtOAc, *n*-Hex) were also found very week irritant, and DCM extract was found moderately irritant. In short, the study concludes that *O. compressa* extracts are safe to use. The cytotoxicity of *Green coffee* nanoemulsions was also investigated via HET-CAM assay [36].

*O. compressa* was studied for hemolytic safety profile. In our results, all the extracts exhibited least hemolytic activity (5.05 ± 0.43 to 8.25 ± 0.83) in comparison to the standard (Triton X-100 = 90.93 ± 1.41). The activity was observed in following order; MtOH > *n*-Hex > EtOAc > DCM > Aq. As a result, all the extracts showed less than 30% hemolytic activity, so it is concluded that plant is nontoxic, non hemolytic and safe. To the best of our knowledge, hemolytic safety profile of *O. compressa* has not been documented previously. Our results are in line with *Portulacaria afra,* reported formerly where the extract was considered dangerous for erythrocytes, if the ratio of hemolysis found greater than 30% [22].

Antioxidant potential of *O. compressa* was evaluated through DPPH assay. All the extracts showed antioxidant activity, especially MtOH extract exhibited significant potential, and the activity was observed in following order; MtOH > Aq > *n-*Hex > EtOAc > DCM. The phytochemical and GCMS analyses of *O. compressa* extracts showed the presence of several compounds such as phenolic, flavonoids and tannins that might be responsible for antioxidant potential. Antioxidants avoid or delay oxidation progression caused by atmospheric oxygen, and play vital role in the defensive mechanism of an organism against pathologies caused by free radicals [37]. Major constituents; alkaloids, flavonoids and tannins act as antioxidant agents reported previously [38].

Earlier studies on *O. compressa* revealed a limited and/or no work against anticancer activity. Therefore, the plant was tested for cytotoxic and anticancer potential against HepG2 cell line through cell viability assays. MTT assay revealed the cytotoxic potential of *O. compressa* extracts in a dose-dependent manner. MTT was followed by calculation of IC_50_ dose; 3 extracts (Aq, MtOH and *n*-Hex) showed IC_50,_ while 2 extracts (DCM and EtOAc) did not showed IC_50_ value. Studies have been reported the anticancer potential of *Kigelia Africana and Dipterygium glaucum* against HepG2 cancer cell line via MTT assay [39,40]. It was observed that *O. compressa* extracts induced the damage of HepG2 cells that later shrink in size. Cancer cells were detached from surface and lost their normal morphology in comparison to control. This condition suggested that cells were in state of toxicity after treatment with plant extracts. It was also noticed that cell viability reduced and rate of cell death was more prominent in treated cells as compared to untreated cells. Cell migration and wound healing potential of *Acacia modesta and Opuntia monocantha* extracts have been documented in liver cancer cell line previously [41]. Presently, it was observed that rate of migration was reduced in treated cells as compared to the untreated; even some extracts disrupted the whole colony of cancer cells. Consequently, the activity was observed in following order; n-Hex > MtOH = Aq = DCM > EtOAc. Findings of the study highlighted a significant anticancer potential of *O. compressa* in damaging HepG2 cancer cells as evidenced by various anticancer assays.

In our GCMS findings, several compounds were identified from *O. compressa* extracts that exhibited the cytotoxicity and anticancer activity against HepG2 cell line. The bis (2-ethylhexyl) phthalate is present in Aq, DCM, MtOH and n-Hex extracts. The methyl tetradecanoate is found in Aq and n-Hex, while n-hexadecanoic acid is present in DCM, EtOH and MtOH extracts. The 11-octadecenoic acid, methyl ester is present in DCM and n-hex extracts, while methyl 18-methylnonadecanoate found in MtOH and *n*-Hex extracts. Moreover, 3-Butoxy-1,1,1,7,7,7-hexamethyl-3,5,5-tris(trimethylsiloxy)tetrasiloxane is present in Aq and DCM extracts, while 4-Vinylphenol is present in MtOH extract of *O. compressa.* These compounds might be potent anticancer agents against HepG2 cell line. Hence, these could be leading candidates for new anticancer drugs. The compounds should be explored against other cancer cell lines as well. Our results are in comparison with the compounds already reported in the previous literature.

The bis (2-ethylhexyl) phthalate was isolated from *Aloe vera* that showed anticancer activity against K562, HL60 and U937 leukemic cell lines [42]. The compound was also reported against MLTC-1 cell line via flow cytometry analysis and genetic studies; and against K562 cell line via MTT assay, caspases pathway, regulating mitochondrial enzymes, Bax mRNA expression, DNA fragmentation and flow cytometric analysis [43,44]. The methyl tetradecanoate was isolated from methanol portion of *Melastomastrum capitatum* which showed anticancer activity against ovarian cancer cell line (OV7) [45]. The n-hexadecanoic acid was found in dry leaf powder extract of *Kigelia pinnata*, reported against human colorectal carcinoma (HCT-116). Based on docking studies, it was observed that cytotoxic activity of n-hexadecanoic acid was due to its interaction with DNA topoisomerase-I [46]. The compounds; n-hexadecanoic acid and methyl tetradecanoate were found in *Sargassum crassifolium* extract, reported against skin melanoma cancer cell line, B16-F10 [47]. The compounds; hexadecanoic acid, and 11-octadecenoic acid, methyl ester were found in *Oscillatoria princeps*, reported against MCF-7, HCT116 and HepG2 cancer cell lines [48]. The 11-octadecenoic acid, methyl ester was also found in Persian Gulf sponge *Axinella sinoxea,* reported against MOLT-4, MCF-7 and HT-29 cancer cell lines [49]. In a recent report, hexadecanoic acid-enriched extract of *Halymenia durvillei* induced cytotoxicity in MDA-MB-231 cells through mitochondrial membrane dysfunction, induction of apoptotic markers and increased expression of LC-3, and also modulated the expression of endoplasmic reticulum stress genes [50]. The 11-octadecenoic acid has been identified from *Capparis decidua* (Forssk) seed oil via GCMS analysis and was predicted as a potent anticancer agent via docking and molecular dynamics studies [51]. The methyl 18-methylnonadecanoate was found in *Amritotharanam kashyam,* reported for cytotoxic potential against MCF-7 cancer cell line via MTT and molecular docking [52]. In the antiproliferative assays, 3-Butoxy-1,1,1,7,7,7-hexamethyl-3,5,5-tris(trimethylsiloxy)tetrasiloxane) was found in ethyl acetate and methanol extracts of *Cissus cornifolia* that exhibited a potent inhibition of the MCF-7–21 cell line, especially methanol extract exhibited potent inhibition of COX-2 and 15-LOX enzymes [53]. Recently, it has been reported that ethanolic extract of Lagerstroemia speciosa (L.) Pers. caused significant concentration-dependent cytotoxicity in HepG2 cells. Studies using flow cytometry and DAPI staining revealed chromatin condensation, a rise in the number of apoptotic cells, and cell cycle arrest at the subG0/G1 phase. The expressions of p-Akt, MDM2, CDK4, cyclin D1, and E1 expressions were down regulated, while expressions of p53, p21, p27, and FOXO1 were markedly up regulated. These results implied that plant causes HepG2 cells to undergo apoptosis and G1-phase cell cycle arrest [54]. In a study, 4-Vinylphenol (4VP) revealed anti-angiogenic effects against HUVEC and HMEC-1 cells and may lead to the inhibition of PI3K/AKT signaling and MMP activation. It may decline cell proliferation and migration/invasion by down regulating the VEGFR expressions [55]. The 4VP has been reported against breast cancer stem-like cells (CSC) where it lead to the inhibition of colony formation, sphere formation, cell proliferation, migration, the expression and activity of ECM-associated proteases that involved in the degradation of extracellular matrix (ECM). It also inhibited the metastasis *in-vivo*. The study suggested the inhibitory actions of 4VP in breast CSC through betacatenin, EGFR and AKT signaling [56].

*O. compressa* was evaluated for thrombolytic potential. In our results, all the extracts showed significant activity, especially a strong thrombolytic potential was observed by EtOAc (96.2 ± 0.88), almost alike to Streptokinase (99.3 ± 0.41) that was evident for the highest clot lysis. The activity was found in following order; EtOAc > DCM > MtOH > *n*-Hex > Aq. To the best of our knowledge, thrombolytic activity of *O. compressa* has not been reported formerly. Several thrombolytic drugs; tissue plasminogen activator (tPA), streptokinase, urokinase, alteplase, and anistreplase, have been used to break up clots [15]. The outcomes of current study are in line with the previous findings [57]. In GCMS findings, several compounds were identified from EtOAc extract of *O. compressa.* These include; polyphenols such as; Heptadecanolide, n-Propyl 9,12,15-octadecatrienoate and 1,2-Benzenedicarboxylic acid dibutyl ester; a fatty acid; pinolenic acid, and a lactone; 4,8,12,16-Tetramethylheptadecan-4-olide. These compounds might be responsible for thrombolytic activity and could be leading agents for new thrombolytic drugs. Our results are in comparison with the compounds reported previously. Polyphenols were present in *Salvia miltiorrhiza,* reported as antithrombotic agents [58]. The dietary fatty acids and Atractylodes lactone found as antithrombotic agents [59,60].

Coagulation factor XI was initially identified as part of the contact pathway of coagulation. However, the traditional theory of the extrinsic and intrinsic pathways has been updated; revealing that factor XI is activated by thrombin and contributes to sustained thrombin generation and the inhibition of fibrinolysis [61]. Molecular docking was performed targeting the active form, FXIa, using all natural compounds identified by GCMS analysis of EtOAc. The results suggested that binding affinity of some compounds was significant as compared to residual compounds. Among them, heptadecanolide demonstrated the strongest binding affinity, with a docking score of 7.3 kcal/mol, suggesting it as a promising lead candidate for further thrombolytic research. For benchmark comparison, we used milvexian, an orally bioavailable and reversible small-molecule FXIa inhibitor, currently undergoing late-stage clinical evaluation. In phase-I trials, milvexian was well-tolerated, induced dose-dependent prolongation of activated partial thromboplastin time (aPTT), and significantly reduced FXI clotting activity [62]. Although heptadecanolide’s 7.3 kcal/mol docking score is weaker than milvexian (often −9 kcal/mol in similar docking setups), its strong interaction signal supports further experimental evaluation to assess its inhibitory potency both *in-vitro* and *in-vivo*. Whereas the residual compounds; 4,8,12,16-Tetramethylheptadecan-4-olide (−5.6 kcal/mol), Pinolenic acid (−5.6 kcal/mol), n-Propyl 9,12,15-octadecatrienoate (−5.3 kcal/mol), and 1,2-Benzenedicarboxylic acid dibutyl ester (−5.3 kcal/mol), exhibited moderate level of binding affinity but lower than heptadecanolide (7.3 kcal/mol) in comparison to milvexian (−9.3 kcal/mol). The best docked compounds were examined further using the web application Swiss ADME, which provided data on their pharmacokinetics, drug similarity and physiochemical characteristics. The results of the ADME analysis revealed that all of the selected compounds are orally suitable and safe to use.

ProTox-II program predicts the toxicity by finding likeness of the chemical structures and comparing with other chemicals having known toxicities (31). Five compounds were chosen from GCMS results of EtOAc extract on the basis of highest binding affinity (with best docking score) and further studied using online PROTOX program for toxicity analysis. The chosen compounds can be subjected to the information about their predicted LD_50_, carcinogenicity, cytotoxicity, hepatotoxicity, immunotoxicity, mutagenicity and predicted toxicity class using ProTox-II online tool. All the phytocompounds showed negative results in hepatotoxicity, mutagenicity, immunotoxicity and cytotoxicity. Only 2 phytocompounds; n-Propyl 9,12,15-octadecatrienoate and 1,2-Benzenedicarboxylic acid, dibutyl ester, showed positive results in carcinogenicity.

## 5 Conclusions

The phytochemical screening and GCMS analysis revealed different phytocompounds in plant extracts. The extracts of *O. compressa* showed non or/ and weak to moderate irritant potentials via HET CAM test. The plant is non-toxic, non-hemolytic and considered safe. The extracts showed significant antioxidant potential in order; MtOH > Aq > *n*-Hex > EtOAc > DCM. The anticancer outcomes of the extracts were found in order; n-Hex > MtOH = Aq = DCM > EtOAc that exhibited significant cytotoxicity against HepG2 cell line showing a strong anticancer potential of *O. compressa*. The plant expressed a major thrombolytic potential as compared with Streptokinase (standard) and provided strong evidence that the herb can be used in treatment of clots. Moreover, *in-silico* molecular docking studies provided more support in favor of EtOAc extract for thrombolytic potential.

## 6 Future Perspectives

*O. compressa* plant can further be evaluated for *in-vitro* and *in-vivo* studies related to cytotoxicity, hemolysis, antioxidant, anticancer and thrombolytic activities. On the basis of encouraging results displayed in liver cancer cell line, the plant may be screened in other cancer cell lines as well. *In-vivo* studies may be conducted in cancer patients. Furthermore, active phytocompounds may be isolated and identified. Possible mechanisms at cellular and molecular level can also be discovered in future.

## Supporting information

S1 File Biosafety ORIC. (JPG)

S2 File Human Ethics ORIC. (JPG)

S3 File Synopsis approval by AS & RB. (JPG)

S4 File Inclusivity-in-global-research-questionnaire. (DOCX)
