# Supplementary figures and images for: Exploring in-vitro antioxidant, cytotoxicity, hemolytic, thrombolytic and anticancer potentials of Ochthochloa compressa (Forssk.) Hilu
Source: PLoS One. 2025 Sep 24;20(9):e0332194. doi: 10.1371/journal.pone.0332194 (PMC12459814; doi:10.1371/journal.pone.0332194)

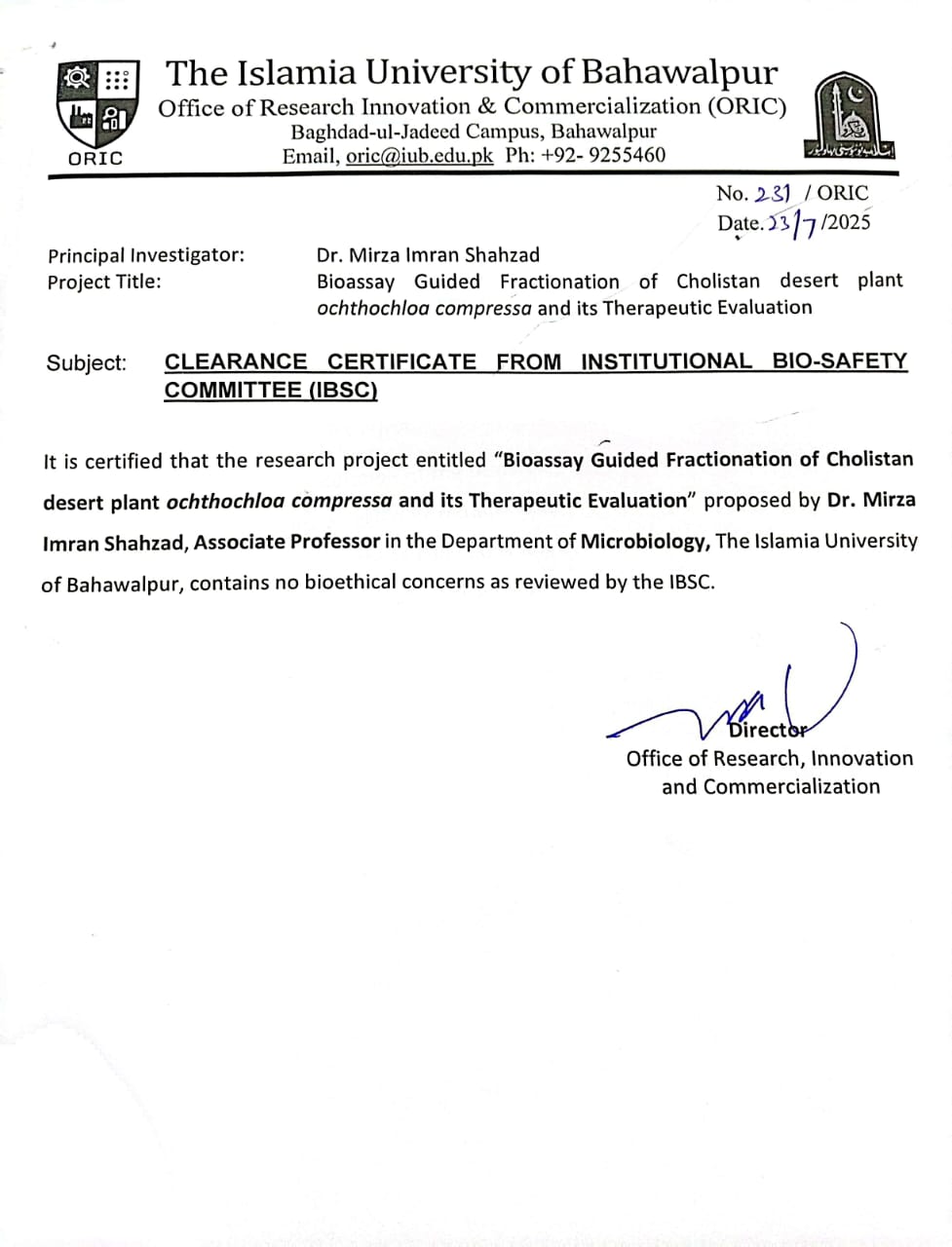

Supplement: S1 File — (JPG) [file pone.0332194.s001.jpg]

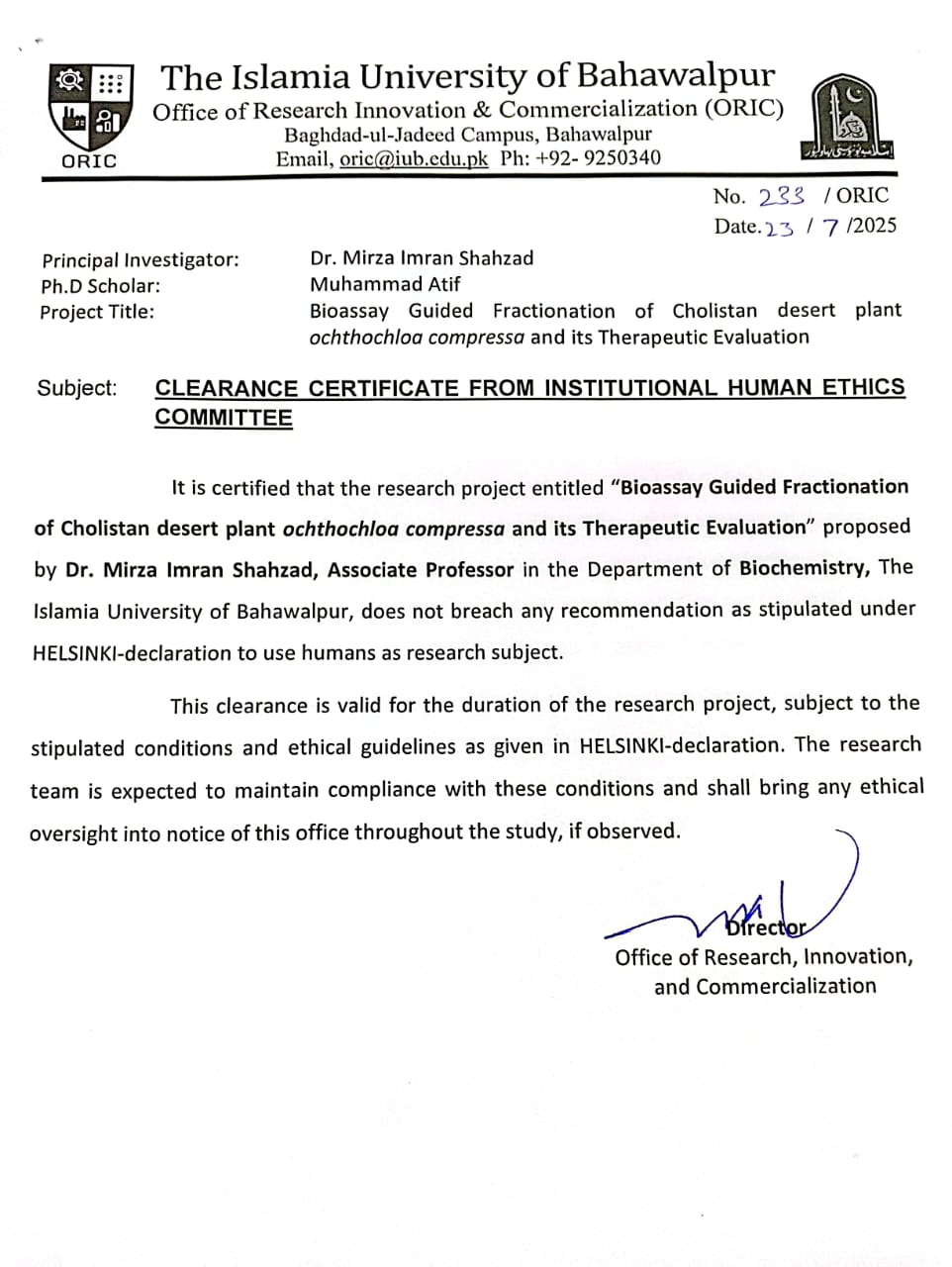

Supplement: S2 File — (JPG) [file pone.0332194.s002.jpg]

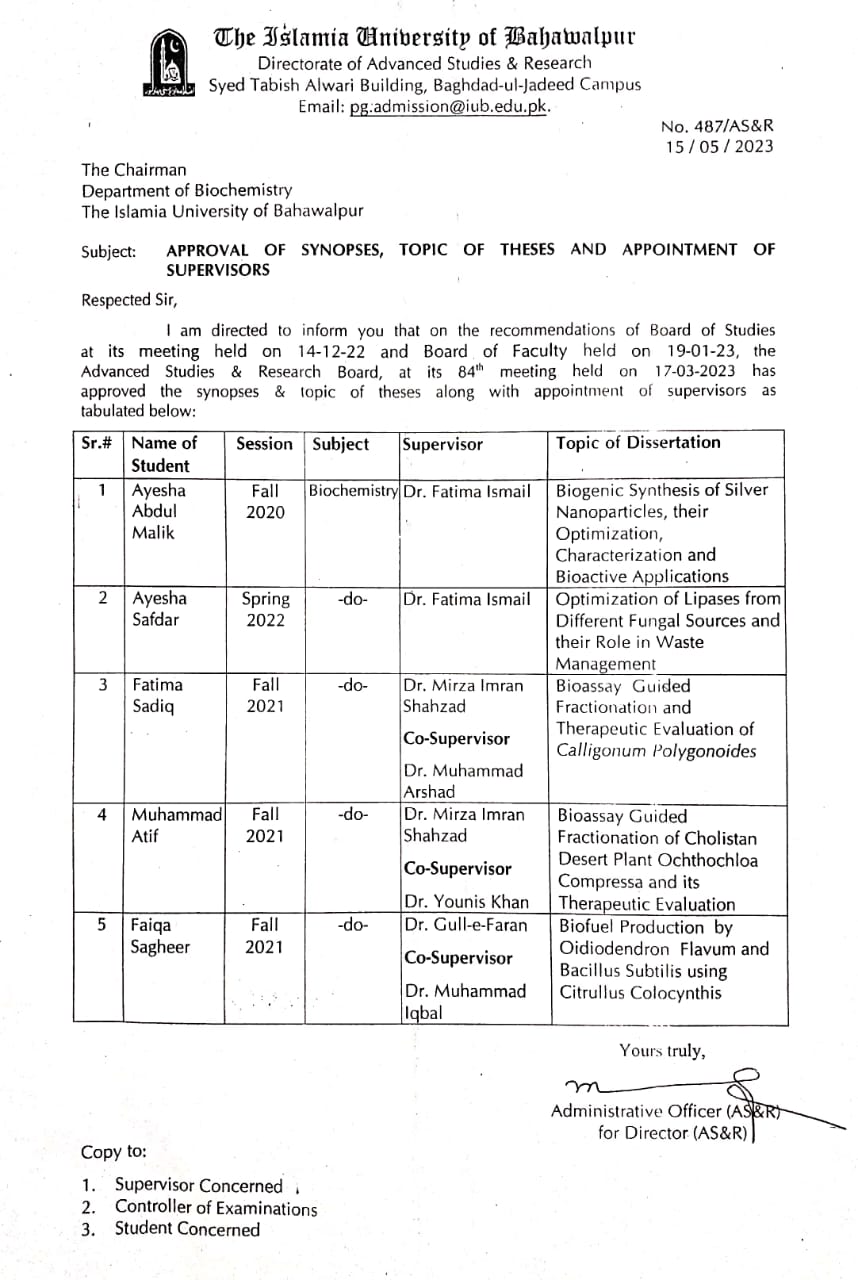

Supplement: S3 File — (JPG) [file pone.0332194.s003.jpg]
